# (2*E*)-3-(3-Bromo­phen­yl)-1-(4-chloro­phen­yl)prop-2-en-1-one: a non-merohedral twin

**DOI:** 10.1107/S1600536809027615

**Published:** 2009-07-18

**Authors:** Hongqi Li, K. Prakash Kamath, B. Narayana, H. S. Yathirajan, William T. A. Harrison

**Affiliations:** a Key Laboratory of Science & Technology of Eco-Textiles, Ministry of Education, College of Chemistry, Chemical Engineering & Biotechnology, Donghua University, Shanghai 201620, People’s Republic of China; b Department of Studies in Physics, Mangalore University, Mangalagangotri 574 199, India; c Department of Studies in Chemistry, Mangalore University, Mangalagangotri 574 199, India; d Department of Studies in Chemistry, University of Mysore, Manasagangotri, Mysore 570 006, India; e Department of Chemistry, University of Aberdeen, Aberdeen AB24 3UE, Scotland

## Abstract

In the title compound, C_15_H_10_BrClO, the mol­ecule adopts an *E* configuration with respect to the C=C double bond and the dihedral angle between the aromatic ring planes is 3.98 (16)°. In the crystal, inversion dimers linked by pairs of C—H⋯O bonds are seen and weak π–π stacking [centroid–centroid separation = 3.8776 (19) Å] may further consolidate the structure. The crystal studied was a non-merohedral twin with a ratio of the twin components of 0.9093 (13):0.0907 (13). The twin operation is a twofold rotation around c*.

## Related literature

For related structures and background to bromo- and chloro-substituted chalcones, see: Yathirajan *et al.* (2006 ▶), Sarojini *et al.* (2007 ▶).
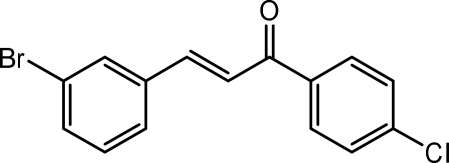


## Experimental

### 

#### Crystal data

C_15_H_10_BrClO
*M* *_r_* = 321.59
Monoclinic, 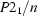

*a* = 14.7421 (10) Å
*b* = 6.1024 (4) Å
*c* = 15.1874 (10) Å
β = 103.905 (2)°
*V* = 1326.25 (15) Å^3^
*Z* = 4
Mo *K*α radiation
μ = 3.28 mm^−1^
*T* = 296 K
0.59 × 0.58 × 0.41 mm

#### Data collection

Bruker SMART CCD diffractometer
Absorption correction: multi-scan (*SADABS*; Bruker, 2001 ▶) *T*_min_ = 0.248, *T*_max_ = 0.346 (expected range = 0.186–0.260)
2609 measured reflections
2609 independent reflections
2273 reflections with *I* > 2σ(*I*)

#### Refinement

*R*[*F* ^2^ > 2σ(*F* ^2^)] = 0.040
*wR*(*F* ^2^) = 0.120
*S* = 1.05
2609 reflections
164 parameters
H-atom parameters constrained
Δρ_max_ = 0.88 e Å^−3^
Δρ_min_ = −0.29 e Å^−3^

### 

Data collection: *SMART* (Bruker, 2001 ▶); cell refinement: *SAINT* (Bruker, 2001 ▶); data reduction: *SAINT*; program(s) used to solve structure: *SHELXS97* (Sheldrick, 2008 ▶); program(s) used to refine structure: *SHELXL97* (Sheldrick, 2008 ▶) and *PLATON* (Spek, 2009 ▶); molecular graphics: *ORTEP-3* (Farrugia, 1997 ▶); software used to prepare material for publication: *SHELXTL* (Sheldrick, 2008 ▶).

## Supplementary Material

Crystal structure: contains datablocks global, I. DOI: 10.1107/S1600536809027615/kj2127sup1.cif

Structure factors: contains datablocks I. DOI: 10.1107/S1600536809027615/kj2127Isup2.hkl

Additional supplementary materials:  crystallographic information; 3D view; checkCIF report

## Figures and Tables

**Table 1:** Hydrogen-bond geometry (Å, °)

*D*—H⋯*A*	*D*—H	H⋯*A*	*D*⋯*A*	*D*—H⋯*A*
C1—H1⋯O1^i^	0.93	2.53	3.328 (4)	144

Symmetry code: (i) 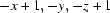.

**Fig. 1.**
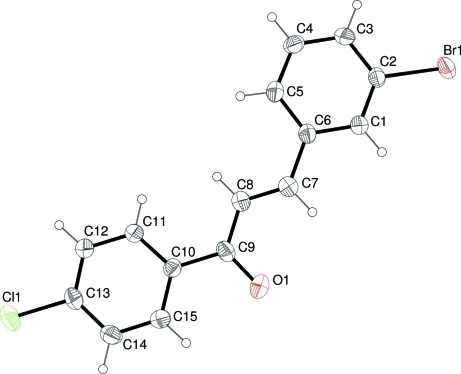
The molecular structure of the title compound with displacement ellipsoids drawn at the 50% probability level and H atoms shown as spheres of arbitrary radius.

**Fig. 2.**
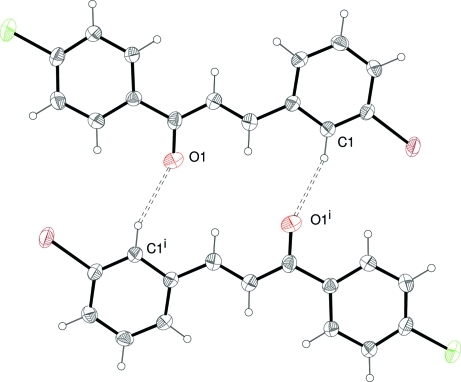
An inversion dimer in the crystal of the title compound linked by two C—H···O bonds (double dashed lines). Symmetry code: (i) 1–x, –y, 1–z.

## supplementary crystallographic information

### Comment

As part of our ongoing studies of bromo- and chloro-substituted chalcones (Yathirajan, *et al.*, 2006; Sarojini, *et al.*, 2007), we now present the synthesis and structure of the title compound (Fig. 1).

The molecule adopts an E configuration with respect to the C=C double bond of the propenone unit. The two benzene rings are approximately coplanar, with a dihedral angle of 3.98 (16)° between their mean planes. The bromine atom is displaced from the C1–C6 mean plane by -0.051 (1)Å and the chlorine atom is displaced from C10–C15 by 0.028 (1) Å. In the crystal, inversion dimers linked by pairs of weak C—H···O interactions (Table 1, Fig. 2) occur.

### Experimental

50% KOH was added to a solution of 4-chloroacetophenone (1.54 g, 0.01 mol) and 3-bromobenzaldehyde (1.86 g, 0.01 mol) in 25 ml of ethanol at 273 K. The mixture was stirred for an hour at room temperature and then poured onto crushed ice. A yellow precipitate was collected by filtration and purified by recrystallization from ethanol. Yellow blocks of the title compound were grown from a mixture of acetone and toluene (1:1) by slow evaporation. Yield of the compound was 80%. Analysis: found (calculated): C%, 55.94 (56.02); H%, 3.10 (3.13).

### Refinement

The H atoms were placed at calculated positions (C—H = 0.93 Å) and refined as riding with *U*_iso_(H) = 1.2*U*_eq_(C). The investigated crystal was a non-merohedral twin with a ratio of the twin components of 0.9093 (13):0.0907 (13) corresponding to a 2-fold rotation about (0 0 1), as determined with the TwinRotMat option of *PLATON* (Spek, 2009). The final refinement was carried out agains a detwinned data set.

### Crystal data

**Table:**

C_15_H_10_BrClO	*F*(000) = 640
*M**_r_* = 321.59	*D*_x_ = 1.611 Mg m^−^^3^
Monoclinic, *P*2_1_/*n*	Mo *K*α radiation, λ = 0.71073 Å
Hall symbol: -P 2yn	Cell parameters from 7751 reflections
*a* = 14.7421 (10) Å	θ = 2.2–28.0°
*b* = 6.1024 (4) Å	µ = 3.28 mm^−^^1^
*c* = 15.1874 (10) Å	*T* = 296 K
β = 103.905 (2)°	Block, yellow
*V* = 1326.25 (15) Å^3^	0.59 × 0.58 × 0.41 mm
*Z* = 4	

### Data collection

**Table:**

Bruker SMART CCD diffractometer	2609 independent reflections
Radiation source: fine-focus sealed tube	2273 reflections with *I* > 2σ(*I*)
graphite	*R*_int_ = 0.0000
ω scans	θ_max_ = 26.0°, θ_min_ = 1.7°
Absorption correction: multi-scan (*SADABS*; Bruker, 2001)	*h* = −18→18
*T*_min_ = 0.248, *T*_max_ = 0.346	*k* = −7→7
2609 measured reflections	*l* = −7→18

### Refinement

**Table:**

Refinement on *F*^2^	Primary atom site location: structure-invariant direct methods
Least-squares matrix: full	Secondary atom site location: difference Fourier map
*R*[*F*^2^ > 2σ(*F*^2^)] = 0.040	Hydrogen site location: inferred from neighbouring sites
*wR*(*F*^2^) = 0.120	H-atom parameters constrained
*S* = 1.05	*w* = 1/[σ^2^(*F*_o_^2^) + (0.0724*P*)^2^ + 0.8199*P*] where *P* = (*F*_o_^2^ + 2*F*_c_^2^)/3
2609 reflections	(Δ/σ)_max_ < 0.001
164 parameters	Δρ_max_ = 0.88 e Å^−^^3^
0 restraints	Δρ_min_ = −0.29 e Å^−^^3^

### Special details

**Table:**

Geometry. All e.s.d.'s (except the e.s.d. in the dihedral angle between two l.s. planes) are estimated using the full covariance matrix. The cell e.s.d.'s are taken into account individually in the estimation of e.s.d.'s in distances, angles and torsion angles; correlations between e.s.d.'s in cell parameters are only used when they are defined by crystal symmetry. An approximate (isotropic) treatment of cell e.s.d.'s is used for estimating e.s.d.'s involving l.s. planes.
Refinement. Refinement of *F*^2^ against ALL reflections. The weighted *R*-factor *wR* and goodness of fit *S* are based on *F*^2^, conventional *R*-factors *R* are based on *F*, with *F* set to zero for negative *F*^2^. The threshold expression of *F*^2^ > σ(*F*^2^) is used only for calculating *R*-factors(gt) *etc*. and is not relevant to the choice of reflections for refinement. *R*-factors based on *F*^2^ are statistically about twice as large as those based on *F*, and *R*- factors based on ALL data will be even larger.

### Fractional atomic coordinates and isotropic or equivalent isotropic displacement parameters (Å^2^)

**Table:**

	*x*	*y*	*z*	*U*_iso_*/*U*_eq_	
C1	0.4051 (2)	0.2640 (5)	0.2830 (2)	0.0443 (7)	
H1	0.4322	0.1261	0.2945	0.053*	
C2	0.3780 (2)	0.3405 (5)	0.1953 (2)	0.0441 (7)	
C3	0.3410 (2)	0.5470 (6)	0.1759 (2)	0.0509 (8)	
H3	0.3226	0.5956	0.1163	0.061*	
C4	0.3316 (2)	0.6805 (5)	0.2464 (2)	0.0504 (8)	
H4	0.3079	0.8214	0.2342	0.060*	
C5	0.3567 (2)	0.6075 (5)	0.3344 (2)	0.0477 (7)	
H5	0.3506	0.7000	0.3813	0.057*	
C6	0.3918 (2)	0.3935 (6)	0.3544 (2)	0.0472 (7)	
C7	0.4073 (2)	0.2956 (6)	0.4461 (2)	0.0515 (8)	
H7	0.4370	0.1601	0.4544	0.062*	
C8	0.3834 (3)	0.3799 (6)	0.5171 (3)	0.0582 (9)	
H8	0.3525	0.5139	0.5112	0.070*	
C9	0.4039 (2)	0.2704 (6)	0.6041 (2)	0.0503 (8)	
C10	0.3899 (2)	0.3888 (5)	0.6857 (2)	0.0422 (7)	
C11	0.3495 (2)	0.5970 (6)	0.6819 (2)	0.0481 (7)	
H11	0.3302	0.6680	0.6264	0.058*	
C12	0.3382 (3)	0.6981 (6)	0.7600 (2)	0.0518 (8)	
H12	0.3104	0.8355	0.7571	0.062*	
C13	0.3684 (2)	0.5938 (6)	0.8414 (2)	0.0523 (8)	
C14	0.4088 (3)	0.3879 (7)	0.8473 (2)	0.0580 (9)	
H14	0.4292	0.3190	0.9032	0.070*	
C15	0.4182 (2)	0.2872 (6)	0.7689 (2)	0.0525 (8)	
H15	0.4442	0.1478	0.7721	0.063*	
Br1	0.39001 (3)	0.15363 (7)	0.09849 (2)	0.06520 (19)	
Cl1	0.35623 (9)	0.7258 (2)	0.93947 (7)	0.0815 (4)	
O1	0.4300 (2)	0.0808 (5)	0.61022 (19)	0.0731 (8)	

### Atomic displacement parameters (Å^2^)

**Table:**

	*U*^11^	*U*^22^	*U*^33^	*U*^12^	*U*^13^	*U*^23^
C1	0.0517 (16)	0.0409 (16)	0.0405 (16)	0.0034 (13)	0.0114 (13)	−0.0024 (13)
C2	0.0526 (17)	0.0432 (16)	0.0401 (16)	−0.0059 (13)	0.0181 (13)	−0.0060 (13)
C3	0.0623 (19)	0.0523 (19)	0.0414 (16)	0.0001 (16)	0.0187 (14)	0.0089 (15)
C4	0.0551 (18)	0.0391 (16)	0.060 (2)	0.0048 (14)	0.0191 (15)	0.0078 (14)
C5	0.0545 (18)	0.0426 (17)	0.0468 (17)	0.0035 (13)	0.0139 (14)	−0.0091 (14)
C6	0.0535 (17)	0.0461 (17)	0.0403 (16)	0.0080 (14)	0.0078 (13)	−0.0028 (13)
C7	0.0591 (19)	0.0495 (18)	0.0469 (18)	0.0030 (15)	0.0145 (15)	−0.0012 (15)
C8	0.071 (2)	0.059 (2)	0.0481 (19)	0.0158 (17)	0.0221 (17)	0.0028 (15)
C9	0.0515 (17)	0.0513 (19)	0.0463 (18)	0.0074 (15)	0.0081 (14)	−0.0043 (14)
C10	0.0420 (15)	0.0409 (16)	0.0451 (16)	−0.0005 (12)	0.0129 (12)	−0.0015 (13)
C11	0.0614 (18)	0.0457 (17)	0.0396 (16)	0.0064 (14)	0.0173 (14)	0.0036 (13)
C12	0.066 (2)	0.0447 (17)	0.0488 (18)	0.0072 (15)	0.0212 (15)	−0.0015 (14)
C13	0.0562 (18)	0.064 (2)	0.0406 (17)	−0.0010 (16)	0.0194 (14)	−0.0062 (15)
C14	0.068 (2)	0.067 (2)	0.0410 (18)	0.0094 (18)	0.0172 (16)	0.0081 (16)
C15	0.0568 (18)	0.0491 (18)	0.0523 (19)	0.0081 (15)	0.0147 (15)	0.0062 (15)
Br1	0.1023 (3)	0.0578 (3)	0.0422 (2)	−0.00177 (19)	0.0304 (2)	−0.00801 (15)
Cl1	0.1037 (8)	0.0997 (9)	0.0472 (5)	0.0144 (7)	0.0302 (5)	−0.0135 (5)
O1	0.102 (2)	0.0526 (15)	0.0671 (17)	0.0213 (15)	0.0250 (15)	−0.0046 (13)

### Geometric parameters (Å, °)

**Table:**

C1—C2	1.377 (5)	C8—H8	0.9300
C1—C6	1.393 (4)	C9—O1	1.216 (5)
C1—H1	0.9300	C9—C10	1.491 (4)
C2—C3	1.377 (5)	C10—C15	1.379 (5)
C2—Br1	1.902 (3)	C10—C11	1.398 (4)
C3—C4	1.379 (5)	C11—C12	1.382 (5)
C3—H3	0.9300	C11—H11	0.9300
C4—C5	1.373 (5)	C12—C13	1.366 (5)
C4—H4	0.9300	C12—H12	0.9300
C5—C6	1.411 (5)	C13—C14	1.384 (5)
C5—H5	0.9300	C13—Cl1	1.740 (3)
C6—C7	1.482 (5)	C14—C15	1.375 (5)
C7—C8	1.317 (5)	C14—H14	0.9300
C7—H7	0.9300	C15—H15	0.9300
C8—C9	1.446 (5)		
			
C2—C1—C6	119.9 (3)	C9—C8—H8	119.0
C2—C1—H1	120.1	O1—C9—C8	120.1 (3)
C6—C1—H1	120.1	O1—C9—C10	120.2 (3)
C3—C2—C1	121.6 (3)	C8—C9—C10	119.7 (3)
C3—C2—Br1	119.3 (2)	C15—C10—C11	118.6 (3)
C1—C2—Br1	119.0 (2)	C15—C10—C9	118.2 (3)
C2—C3—C4	118.9 (3)	C11—C10—C9	123.2 (3)
C2—C3—H3	120.5	C12—C11—C10	120.5 (3)
C4—C3—H3	120.5	C12—C11—H11	119.7
C5—C4—C3	120.7 (3)	C10—C11—H11	119.7
C5—C4—H4	119.6	C13—C12—C11	119.2 (3)
C3—C4—H4	119.6	C13—C12—H12	120.4
C4—C5—C6	120.6 (3)	C11—C12—H12	120.4
C4—C5—H5	119.7	C12—C13—C14	121.5 (3)
C6—C5—H5	119.7	C12—C13—Cl1	118.7 (3)
C1—C6—C5	118.1 (3)	C14—C13—Cl1	119.7 (3)
C1—C6—C7	118.9 (3)	C15—C14—C13	118.7 (3)
C5—C6—C7	122.7 (3)	C15—C14—H14	120.6
C8—C7—C6	126.9 (3)	C13—C14—H14	120.6
C8—C7—H7	116.5	C14—C15—C10	121.4 (3)
C6—C7—H7	116.5	C14—C15—H15	119.3
C7—C8—C9	122.0 (3)	C10—C15—H15	119.3
C7—C8—H8	119.0		
			
C6—C1—C2—C3	2.5 (5)	O1—C9—C10—C15	7.9 (5)
C6—C1—C2—Br1	−176.2 (2)	C8—C9—C10—C15	−174.1 (3)
C1—C2—C3—C4	0.5 (5)	O1—C9—C10—C11	−172.1 (3)
Br1—C2—C3—C4	179.3 (3)	C8—C9—C10—C11	5.9 (5)
C2—C3—C4—C5	−1.4 (5)	C15—C10—C11—C12	−0.2 (5)
C3—C4—C5—C6	−0.7 (5)	C9—C10—C11—C12	179.8 (3)
C2—C1—C6—C5	−4.5 (5)	C10—C11—C12—C13	1.1 (5)
C2—C1—C6—C7	170.4 (3)	C11—C12—C13—C14	−1.0 (6)
C4—C5—C6—C1	3.6 (5)	C11—C12—C13—Cl1	178.5 (3)
C4—C5—C6—C7	−171.1 (3)	C12—C13—C14—C15	−0.2 (6)
C1—C6—C7—C8	−167.8 (4)	Cl1—C13—C14—C15	−179.6 (3)
C5—C6—C7—C8	6.9 (6)	C13—C14—C15—C10	1.2 (5)
C6—C7—C8—C9	−178.8 (3)	C11—C10—C15—C14	−1.0 (5)
C7—C8—C9—O1	−12.1 (6)	C9—C10—C15—C14	179.0 (3)
C7—C8—C9—C10	169.8 (4)		

### Hydrogen-bond geometry (Å, °)

**Table:**

*D*—H···*A*	*D*—H	H···*A*	*D*···*A*	*D*—H···*A*
C1—H1···O1^i^	0.93	2.53	3.328 (4)	144

Symmetry codes: (i) −*x*+1, −*y*, −*z*+1.
